# Error in Text

**DOI:** 10.1001/jamanetworkopen.2018.5010

**Published:** 2018-10-12

In the Invited Commentary titled “Death, Readmissions, and Getting Policy Right,”^1^ published on September 28, 2018, there were errors in the text. In the fourth paragraph, the first sentence should read, “First, for 2 of the 3 conditions, the rate of decrease of inpatient mortality slowed significantly after the HRRP announcement, during the same time in which readmissions decreased. They appear to have resumed at the same rate during implementation of HRRP penalties.” In the fifth paragraph, the first sentence should read, “The changes in inpatient mortality during the announcement phase are of interest.” and the third and fourth sentences should read “With the announcement of the HRRP, something appeared to change: the improvement in inpatient mortality rates for both conditions slowed significantly. How might the HRRP explain this?” In the sixth paragraph the first sentence should read “The findings by Khera et al^5^ and those by Gupta et al^3^ (who found an increase in mortality after HRRP) raise an important issue: what happens with inpatient mortality rate affects what happens during the postdischarge period. Inpatient mortality, by definition, shapes the population of patients who are discharged and eligible to be readmitted. If there is a change in inpatient mortality—even if there is simply a slowing of the long-term gains in mortality—the patient population discharged alive will be healthier than they would have been otherwise.” This article has been corrected.^1^
